# Hepatitis B Vaccination, Knowledge, Attitudes, and Practices Among Sample of Greek Nursing Students: A Cross-Sectional Study

**DOI:** 10.3390/nursrep14040234

**Published:** 2024-10-28

**Authors:** Anastasia Statiri, Theodoula Adamakidou, Ourania Govina, Nikoletta Margari, Eugenia Vlachou, Eleni Dokoutsidou

**Affiliations:** Department of Nursing, University of West Attica, 12243 Athens, Greece; thadam@uniwa.gr (T.A.); ugovina@uniwa.gr (O.G.); nmargari@uniwa.gr (N.M.); evlachou@uniwa.gr (E.V.); edokout@uniwa.gr (E.D.)

**Keywords:** hepatitis B, nursing students, vaccine uptake, knowledge, attitudes, practices, Greece

## Abstract

Background: Nursing students share their study time between clinical and university environments, and due to their clinical training, are at increased risk of contracting the hepatitis B virus (HBV). This study investigated the vaccination coverage, knowledge, attitudes, and practices of nursing students regarding HBV in Greece. Methods: A cross-sectional study was conducted from September 2022 to July 2023, using an anonymous self-administered questionnaire, with informed consent obtained from each participant. Results: A total of 1261 nursing students from University Institutions of the Attica region participated in the convenience sampling. The response rate of the nursing students was 68.6%. A total of 27.7% of the sample was fully vaccinated against HBV. The reasons for not accepting the vaccine were not found to be related to participants’ level of knowledge or attitudes (*p* > 0.05). The knowledge score ranged from 0% to 96.9%, with a mean of 62.2% (SD = 17.0%). Multivariate linear regression showed that longer year of nursing education was associated with better practices and attitudes towards HBV (*p* < 0.05). Conclusions: The emergence of low vaccination coverage of nursing students against HBV identifies the need for departments of nursing studies to proceed in the development of educational and intervention programs on infection control. This study was not registered.

## 1. Introduction

High vaccination coverage is a key measure of successful public health interventions, protecting individuals and communities from preventable diseases like hepatitis B [1]. The international vaccination goal against the hepatitis B virus (HBV) is to achieve 100% vaccination in all countries by 2030, according to the World Health Organization (WHO) [2]. The development of a safe HBV vaccine in the 1980s contributed to transforming hepatitis B from a fatal disease to a chronic one [3]. Unfortunately, HBV still imposes a major public health problem, with an increased risk of occupational exposure among unvaccinated health professionals [4].

Nursing students share their study time between university and clinical environments, are in proximity to patients, handle bodily fluids, lack experience and knowledge, and therefore are at risk for exposure to HBV and other infections [5]. While clinical settings administrators are not required to check student vaccinations, the university bears the primary responsibility for student safety. They should put measures in place to ensure that students are vaccinated before starting clinical rotations. Additionally, nursing students, like all healthcare students, should follow the vaccination recommendations that apply to health professionals; therefore, vaccination against HBV is mandatory for all of them [4]. A Turkish research study reported that most of the participating nursing students had been fully vaccinated against HBV. However, 28.1% of them had sustained a needle-stick accident and 5.4% had experienced conjunctival exposure to patients’ bodily fluids [6]. Other studies refer to an immunization rate against HBV as high as 6.7–37.2% [7,8,9,10,11,12,13,14], which is three to ten times lower than the WHO’s vaccination target to reach 100% among health professionals [15]. The HBV serology for hepatitis B infection was investigated in a study conducted in South Korea among 2000 university students. The study found that the levels of anti-HBs tested in the students were <10 mIU/mL, 10–100 mIU/mL, and >100 mIU/mL. A titer of <10 mIU/mL indicates that a person is not adequately protected and may need a booster dose of the vaccine [10].

Several factors seem to affect the general level of knowledge, attitudes, and practices of nursing students regarding hepatitis B. Firstly, the level and quality of education and training on hepatitis B provided to nursing students from University Institutions can significantly impact their knowledge and understanding of the disease, as well as their attitudes and practices towards prevention and management [2,6,8,13]. Stigma and discrimination associated with hepatitis B can affect nursing students’ attitudes towards patients with the disease and their willingness to provide care and support to affected individuals [9,10]. Another important factor is the availability of resources, such as educational materials, training programs, and vaccination services. Those can impact nursing students’ ability to acquire knowledge about hepatitis B and implement preventive practices in their clinical practice [2,8]. Interactions with peers and colleagues, and the culture within the nursing school or healthcare facility, can shape nursing students’ attitudes and practices towards hepatitis B, including their willingness to discuss the disease and seek help when needed [4,16]. Moreover, the presence of a national immunization program, the institutional policies, and the guidelines related to hepatitis B screening, vaccination, and infection control can influence nursing students’ adherence to best practices and standards of care in healthcare settings [2,6,13]. Nursing students’ personal beliefs, values, and cultural background can also play a role in shaping their attitudes and practices towards hepatitis B, including their views on vaccination, risk perception, and patient care [4,5].

Hepatitis B vaccination policies vary among European countries and different categories of health professionals, including nursing students. European countries belonging to the Mediterranean basin, such as Greece and Italy, belong to the areas of intermediate HBV endemicity (2–7.9%) [17]. This necessitates the control of HBV infection through early vaccination and the implementation of educational programs in nursing schools. As far as Italy and Greece are concerned, HBV vaccination is recommended for student nurses and other healthcare professionals [18]. However, both countries have an obligatory HBV vaccination policy for newborns. This seems to be the reason for the high vaccination coverage of the adult population found in the studies of Verso et al. (2020) and Papagiannis et al. (2016) [16,18].

The only published study from another Mediterranean country, Cyprus, back in 2019, investigated the vaccination status, knowledge, and behaviors of healthcare students at a Cypriot university regarding HBV. While the high vaccination rates (92.2%) are encouraging, the identified knowledge gaps and areas for improvement highlight the importance of ongoing efforts to enhance HBV education and promote preventive practices among future healthcare professionals [19]. Iraq established a national plan in 2002 to control the spread of hepatitis B but does not offer a mandatory newborn vaccination policy. Although the study suggests high overall vaccination coverage, it identifies limitations in preventing mother-to-child transmission. This highlights the crucial role of an obligatory HBV vaccination policy for newborns [20].

Greece has a robust national immunization program that includes hepatitis B vaccination. The program targets infants, children, and adolescents, as well as health professionals and individuals at high risk of exposure to the virus. A person is considered fully vaccinated against hepatitis B when they have received three doses of a hepatitis B vaccine and at least six months have passed since the third dose of the vaccine. It is important to note that vaccination schedules may vary slightly, depending on the specific vaccine used and the individual’s circumstances. Hepatitis B vaccines are widely available throughout Greece, both in public and private healthcare settings. They are prescribed by physicians when needed and physicians are the first level of surveillance for the disease.

Nursing education in Greece is typically a 4-year undergraduate program. This program is only offered by universities and upon completion, graduates are awarded a Bachelor of Science in Nursing (BScN) degree. The absence of an organized vaccination program for nursing students’ control, vaccination, and surveillance against hepatitis B in Greece, coupled with the mobility of students between practice sites, highlights the need for immediate intervention by Greek departments of nursing studies. Since 2016 there has been a gap in the Greek literature regarding HBV vaccine uptake in nursing students, probably because the last published study revealed a rather promising coverage against the disease (81.4%) [16].

Until today, a great amount of research has investigated the HBV vaccination coverage of health professionals, such as medical and nursing staff, but there is a paucity of research regarding the immunization status of nursing students. Considering the need for up-to-date data about HBV vaccination coverage among nursing students across the world, the present study aimed to determine the vaccination coverage, knowledge, attitudes, and practices of nursing students against hepatitis B in Greece. Further objectives of the present study were as follows: (1) to investigate whether socio-demographic characteristics of nursing students and the year of study affect their vaccination coverage regarding hepatitis B; (2) to study any possible relationship between the level of knowledge, attitudes, and practices of nursing students and their HBV vaccine uptake; and (3) to determine whether the level of knowledge regarding hepatitis B is associated with nursing students’ attitudes and practices.

## 2. Materials and Methods

### 2.1. Study Design

From September 2022 to July 2023, a comprehensive study was carried out involving nursing students from the University of West Attica (UNIWA) and the National and Kapodistrian University of Athens (UOA), which are the two largest University Institutions in Greece. The study was conducted as a cross-sectional analysis at a single point in time. To be eligible for participation, individuals had to be enrolled in a Department of Nursing program and provide informed consent.

### 2.2. Study Participants

The study welcomed participants of any gender, race, ethnicity, or health condition, as long as they identified themselves as adults aged 17 or older. Proficiency in the Greek language was also a requirement for inclusion. All students, regardless of any chronic or acute medical conditions, were included in the study. Students with insufficient knowledge of Greek, and individuals from non-Nursing departments were excluded. A total of 1838 students met the inclusion criteria and were invited to participate in the study. Of these, 1261 questionnaires were returned fully completed, resulting in a response rate of 68.6%. Any questionnaires with missing data were excluded from the analysis.

### 2.3. Data Collection

The main author approached the students in the classrooms of compulsory courses, using a convenience sampling method. Permission was sought from the chairman of the nursing department and then from the professor of each course. The lead author approached the students to provide them with information about the study’s objectives, the voluntary nature of participation, the option to withdraw at any point, the assurance of data confidentiality, and the requirement of consent prior to completing the questionnaire. The data were protected, and each student could access the questionnaire only once. Following their informed consent, the students proceeded to fill out the self-report questionnaire, which was distributed in paper format to nursing students in both universities. Students completed the questionnaire independently and all fully completed questionnaires were collected and digitized by the research team using SPSS statistical software. The students filled in the questionnaire at the end of the course and the departure of the teaching professor so that it is not considered that their participation in the research will affect the judgment of the professor towards them and/or their score.

### 2.4. Questionnaire Development

The survey tool was a structured questionnaire, created for the purpose of this study, and based on data from the international scientific literature. More specifically, a literature review was conducted on students’ knowledge, attitudes, and practices regarding HBV vaccination, along with other relevant assessment tools [8,19,20,21,22]. The valuable expertise of researchers and professors specializing in communicable diseases, infection prevention, public health, community nursing, and statistical science, along with insightful discussions with students, played a significant role in the creation of the questionnaire. The initial questionnaire consisted of 100 questions. To ensure its clarity and appropriateness, an expert panel comprising four nursing professors (T.A., O.G., N.Μ., and E.D.) and four nursing specialists in infection prevention and management were briefed on the purpose of the tool. After carefully reviewing the questionnaire, they provided feedback, categorizing each item as “essential”, “useful but inadequate”, or “unnecessary”. Taking all expert comments into consideration, a total of 72 items were included in the final questionnaire, with the guidance of a statistical scientist. Following the drafting phase, a pilot test was conducted.

The final questionnaire included socio-demographic characteristics, general attitudes toward vaccination, and, in particular, knowledge, attitudes, practices, and vaccination coverage regarding the hepatitis B virus.

More specifically, the demographic characteristics of the participants included 13 questions on gender, age, religion, nationality, marital and employment status, insurance capacity, place of permanent residence, university, year of study, and smoking and alcohol consumption habits. This section of the questionnaire utilized a multiple-choice response format.

The questionnaire included nine questions to assess students’ general attitudes towards vaccinations. Five of these questions focused on their perceptions of vaccine effectiveness against specific diseases like pertussis and measles. The remaining four questions used a 5-point Likert scale to measure their trust in government health information and their reasons for accepting or declining vaccinations.

The questionnaire was followed by 22 questions about the HBV, and more specifically, 1 question received answers in the form of multiple choice (i.e., “What is the international abbreviation for hepatitis B virus?”), and 21 questions were structured to elicit a ternary response set, comprising “yes”, “no”, and “don’t know/don’t answer” as possible answers (i.e., “Are infected individuals asymptomatic during the acute phase of infection?”).

Responses to all 7 inquiries pertaining to students’ perspectives on HBV were collected using a 5-point Likert scale, such as the question “Should hepatitis B patients be isolated?”.

The examination of participants’ behaviors in relation to the disease involved a series of 13 questions. Questions were designed to gather information about previous exposures, preventative practices, and perceived risk. Participants responded to questions requiring a “yes,” “no,” or “don’t know/don’t answer” answer to determine their history of exposure to hepatitis B, including needle-stick injuries and previous testing. A 5-point Likert scale was used to measure attitudes and practices, such as avoidance of hepatitis B patients. Additionally, multiple-choice questions were used to gather information about exposure frequency and preventative behaviors.

The final section of the survey contained a series of 8 inquiries pertaining to the immunization status of the students. Participants were required to indicate whether or not they had received the HBV vaccine. In the event of an affirmative response, they were then prompted to specify the number of vaccine doses they had received through a multiple choice question. The estimated time needed to complete the questionnaire ranged from 15 to 20 min.

### 2.5. Pilot Study

After the development of the questionnaire, it underwent a thorough examination to ensure its reliability and validity. To begin, a pilot test was conducted on a group of 30 nursing students who were not included in the final sample of the study. The research tool’s reliability was assessed using Cronbach’s alpha coefficient and test–retest analysis, resulting in a coefficient of 0.723 for the entire questionnaire. The internal reliability for each of the three sections of the tool (knowledge, attitudes, and practices) exceeded 0.70, while the test–retest reliability for these sections was statistically significant at *p* < 0.05, indicating that the instrument exhibited strong stability.

### 2.6. Study Variables

The dependent variable, HBV vaccination status, was categorized as fully vaccinated, partially vaccinated, or not vaccinated. Independent variables included socio-demographic characteristics (age, gender, year of study), knowledge of HBV (calculated as a percentage of correct answers), and attitudes and practices towards HBV (measured using Likert scale items).

### 2.7. Statistical Analysis

Data were tabulated using the SPSS 26.0 statistical package (SPSS Inc., Chicago, IL, USA). Missing values were reported for any items of the research tool that were not fully completed. To ensure data quality, only questionnaires with complete responses to all critical items were included in the final dataset. A multiple logistic regression analysis was conducted to identify factors associated with HBV vaccination status. A stepwise method was employed with a *p*-value for entry set at 0.05 and a *p*-value for removal set at 0.10. Adjusted odds ratios (ORs) with 95% confidence intervals (CIs) were computed to quantify the strength and direction of the associations between independent variables and HBV vaccination. Variables were presented as counts and proportions. Quantitative variables were expressed as mean values (Standard Deviation) and as median (Interquartile Range), while categorical variables were expressed as absolute and relative frequencies. Also, linear regression analysis was performed in a stepwise method in order to find factors associated with knowledge scores regarding HBV. Internal consistency reliability was checked with the use of Cronbach’s coefficient. All reported *p* values were two-tailed and statistical significance was set at *p* < 0.05.

To ensure the validity of the regression analysis, we checked for the following assumptions. First of all, independence of errors as residuals were examined for any patterns or correlations to assess independence. As far as multicollinearity is concerned, the variance inflation factor (VIF) was used to identify multicollinearity among independent variables. Diagnostic plots, such as scatter plots and residual plots, were used to identify outliers and influential observations. Hosmer and Lemeshow test was used to test goodness of fit of logistic regression model.

### 2.8. Ethics

In accordance with the guidelines set forth in the Declaration of Helsinki, the study was carried out and received approval from the Research Ethics Committee at the University of West Attica (protocol code 17432/23-02-2022). All participants provided informed consent prior to their involvement in the study. An introductory note was provided to the participants, outlining the importance of maintaining anonymity throughout the questionnaire. Only after obtaining written consent were the participants able to complete the self-report questionnaire.

## 3. Results

### 3.1. Socio-Demographic Characteristics

A total of 1838 individuals were invited to participate in the study. Questionnaires were distributed to these individuals, with 1261 being fully completed and returned. Incomplete questionnaires, including those with missing data or partially completed sections, were excluded from the analysis. This resulted in a response rate of approximately 68.6%. The mean age of the participants was 21.9 years (SD = 5.6 years). Moreover, the majority of the participants were Greeks (93.2%), Christians (88.8%), singles (87.8%), and unemployed (61%). Furthermore, most students (28.3%) were in their third year of studies, while 24.3% were smokers and 42.1% consumed alcohol. More than half of the sample (63.3%) trusted/trusted completely the reliability of the information provided by the Ministry of Health about vaccinations. The participants’ main characteristics are presented in Table 1.

### 3.2. Vaccination Coverage Against HBV

The declaration of vaccination against HBV was 66.3%, as 835 out of 1261 nursing students reported vaccination against HBV. However, only 27.7% (N = 349) of the sample were fully vaccinated against the disease. A total of 478 nursing students (37.9%) were partly vaccinated with one (N = 94, 11.4%) or two (N = 384, 46.4%) doses of the vaccine. Only 17.6% (N = 222) of the study population had done an antibody test at least once and most of them (N = 111, 50.5%) declared to have been tested more than a year ago. Half of the nursing students who declared to have been checked for anti-HBs (N = 111, 50.5%) claimed that they did it out of interest for their antibody condition and only seven students (3.2%) were checked because they were sick.

### 3.3. Participants’ Level of Knowledge Regarding HBV

Regarding participants’ level of knowledge on HBV, the percentage of correct answers ranged from 26.7% to 95.9%. More specifically, 26.7% (N = 336) of the sample answered correctly that hepatitis B is diagnosed with the ELISA test and 29.7% (N = 374) answered correctly that hepatitis B is not transmitted by the use of a contaminated toilet. Also, the vast majority of the sample answered correctly that hepatitis B is transmitted by contact with contaminated blood (N = 1208, 95.9%) and that there is a vaccine for the hepatitis B virus (N = 1155, 91.7%). All correct answers were added, and their sum was converted into a percentage. Thus, students’ knowledge scores could range from 0% to 100%, with greater values indicating a higher level of knowledge. Knowledge scores ranged from 0% to 96.9%, with the mean value being 62.2% (SD = 17.0%). Cronbach’s alpha reliability index was 0.83, indicating the acceptable reliability of the score. More information regarding participants’ level of knowledge on HBV can be found in Table 2.

### 3.4. Participants’ Attitudes and Practices on HBV

Table 3 presents participants’ attitudes and practices towards HBV, as measured by a 5-point Likert scale (1 = strongly disagree, 5 = strongly agree). A higher score indicates a more positive attitude or practice. More specifically, there was greater agreement regarding the opinion that newborns of mothers with hepatitis B should be vaccinated against the virus, with the mean value for this question being 4 (SD = 1.3 points), while high was also the agreement with the fact that physical exercise should be allowed in patients with hepatitis B, for which the mean value was 3.9 (SD = 1.2 points). Moreover, 209 out of 252 injured nursing students (83.2%) reported to their supervisor that they had a needle-stick injury, but the rest hid the accident. High agreement was also found between using clean gloves and handling patients’ bodily fluids. The main reason for that was students’ security anxiety (80.6%).

Table 4 shows that compared to male participants, female nursing students were 1.57 times more likely to be fully vaccinated against HBV (*p* = 0.011). No other socio-demographic factor, except gender, was found to be related to vaccine uptake. Multiple logistic regression revealed that students in their third year of nursing studies were more likely to be fully vaccinated against HBV compared to students in their first year of studies (*p* = 0.011). Additionally, the analysis found a significant association between a higher level of knowledge about HBV and a greater probability of being fully vaccinated against the disease (*p* < 0.001). Some participants’ attitudes were significantly correlated with HBV vaccine acceptance. More specifically, students who strongly agreed that patients with hepatitis b should be allowed to work were 14% more likely not to be vaccinated against HBV (*p* = 0.002). Regarding practices of nursing students against the disease, participants who considered the hospitalization of HBV-positive patients a problem had a 13% greater probability of being fully vaccinated against HBV (*p* = 0.047). Although a needle-stick injury is considered bad practice against HBV and other blood-borne diseases, previous injuries were significantly associated with vaccination (*p* = 0.013).

The score for the students’ recommended practices and attitudes towards hepatitis B was 63.8 (SD = 16.5 points). A positive correlation was found between the attitude/practice score for the disease and the overall knowledge score for HBV (*p* < 0.001).

In order to highlight the factors independently related to the attitudes and practices of nursing students regarding hepatitis B, a multivariate linear regression in a stepwise method was performed, as presented in Table 5.

As presented in Table 5, nursing students in their second year of study were found to have significantly different HBV attitudes and practices scores compared to students in their first year of study. More specifically, those who were in their second year of study had more appropriate attitudes and practices towards hepatitis B compared to those who were in their first year. Also, higher knowledge scores about hepatitis B implied more appropriate attitudes and practices towards the disease.

## 4. Discussion

This research investigated the level of knowledge, attitudes, and practices against HBV vaccination among Greek nursing students. The results of the present study highlighted the need for further investigation into HBV vaccination practices among Greek nursing students, as current policies may not adequately address the issue. Greece is one of the European countries belonging to the areas of intermediate HBV endemicity (2–7.9%) [17]. To our knowledge, this was the first study that estimated HBV vaccine uptake among nursing students in Greece. In addition to this, the survey provided a wealth of data on the knowledge, attitudes, and practices of the study population regarding HBV. The present study was cross-sectional and used a self-report questionnaire, as most published studies with similar objectives [4,5,6,7,8,9,10,11,12,13,14,23,24,25,26,27]. In the literature, such a large sample size of nursing students has not been previously reported, not only in Greece, but also in any other European country. Only one multicenter study in Turkey managed to enroll 1491 participants [6]. Data from European countries, such as Italy, show a strong research interest in vaccination coverage of health science students. However, the published, multicenter studies of Verso et al. involved a sample of 520 nursing students [18]. In Greece, the only published study on the vaccination status against HBV was that of Papagiannis et al. (2016), in which 716 nursing students participated [16].

However, this previously published Greek study did not clarify whether the high vaccination coverage of 81.4% referred to nursing students who had completed the full hepatitis B vaccination regimen or were only partially vaccinated [16]. Therefore, we cannot safely compare the fully vaccinated rate found in our study (27.7%) with the previously published rate. Other published studies from areas of intermediate HBV endemicity (2–7.9%) retrieved a vaccine uptake between 8.5% [10] and 100% [18]. In Turkey, children born after 1998 received the HBV vaccine as part of a routine vaccination program [7]. Maybe that is the main reason for the high HBV vaccination coverage of nursing students, which reached 85.3% in 2011 [6]. Two studies from India revealed a lower vaccination coverage than our findings, 8.5% in 2015 [10] and 17.6% in 2020 [14]. A study from Nepal found 91.8% fully vaccinated nursing students [28], and in Iran, 412 nursing students (97.2%) had received full HBV vaccination [24]. In Pakistan, another area of intermediate HBV endemicity, nursing students receive the HBV vaccination at the beginning of their clinical rotations [8]. However, due to a limited budget, the vaccination coverage of nursing students remains low (37.2%) [15] but is still higher than our findings. As far as Italy is concerned, a total of 483 nursing students from University of Palermo were fully vaccinated (100%), but 254 of them (52.5%) had a titer of anti-HBsAg lower than 10 mIU/mL [18].

In our research, 81.8% (N = 1032) of the study population had never performed an antibody test for hepatitis B. However, half of those who were at least once checked for anti-HBsAg (N = 222, 17.6%) claimed that they did it out of interest for their antibody condition (N = 111, 50.5%), and only seven students (3.2%) were checked because they were sick. It should be emphasized that, in Greece, no intervention program has been implemented to screen students’ pre- or post-vaccination immunity. Considering that 10% of adults vaccinated with the full regimen against HBV do not develop an anti-HBsAg titer above 10 mIU/mL, it is necessary to check the antibody status and revaccinate those in need [18]. This was implemented in a tertiary academic hospital in South Africa, and the findings showed that only 11% of the vaccinated health professionals who participated in the post-vaccination immunity screening had developed protection against HBV [29]. The cohort studies from Italy showed that vaccination during infancy compared to vaccination during adolescence offers different immunological responses, with higher anti-HBsAg titers observed among nursing students who were vaccinated during adolescence [18,30]. These findings, combined with the fact that Greece, like all countries of the Mediterranean basin, belongs to the areas of intermediate endemicity of HBV, intensifies the need for immediate and drastic interventions.

Another important finding of the study was that the level of knowledge of the participants was found to be positively related to vaccination acceptance (*p* < 0.001). On the same page, a recent study from Ethiopia identified a lack of knowledge about hepatitis B as an important reason for the non-vaccination of nursing students [13]. Similarly, a cross-sectional study from Pakistan, which belongs to the countries with intermediate HBV endemicity like Greece, showed that the level of knowledge of nursing students regarding hepatitis B is significantly (*p* < 0.05) associated with the acceptance of HBV vaccination [8]. Similarly, in the present study, a higher knowledge score, and therefore more knowledge about hepatitis B, meant that participants were more likely to be fully vaccinated (*p* < 0.001).

Nursing students come into contact with the subject of infection and infection control throughout their studies, and over the academic years, they enrich the knowledge acquired in previous years of study. This explains the important finding of this study that those in the third year of nursing studies were 1.69 times more likely to have been fully vaccinated for hepatitis B compared to those in the first year (*p* = 0.011), while those in the second year of studies had more appropriate attitudes and practices towards hepatitis B compared to those in the first year of nursing studies (*p* < 0.05). After multivariable analysis, academic years, good attitudes, and practices were also significantly associated with the full-dose vaccination status of nursing students at Wolkite University of Southwest Ethiopia (*p* < 0.01) [2]. A recent study by a medical training college in Kenya revealed that greater years of nursing studies is associated with a higher prevalence of HBV vaccination (*p* < 0.001) [13]. The value of university education for nursing students is also confirmed by a cross-sectional study in Turkey, where the vast majority of the participants (93.7%) reported that their main source of knowledge about HBV infection was their university education [6]. Mengal et al. (2008) did not find any correlation between the prevalence of HBV vaccination and the year of nursing studies [8].

Of the 1261 students who participated in this study, 20% (N = 252) reported having been injured at least once with a needle. A similar study from Taiwan showed a threefold increase in the rate of needle-stick injuries during nursing students’ practical training (61.9%) [5]. Turkish nursing students also seem to have higher accident rates (28.1%) than Greeks [27]. Nursing students who participated in our survey and had been injured with a needle were 1.55 times more likely to have been fully vaccinated against hepatitis B, compared to those who had not had a similar accident (*p* = 0.013). However, the ultimate aim is the prevention and early vaccination of students before the start of their clinical practice, where the risk of needle-stick injury rapidly increases.

## 5. Limitations

The present study was a cross-sectional survey. Therefore, the data collected refer to a specific moment in time, the moment of completing the survey questionnaire, which is essentially a moment of capturing the knowledge, attitudes, and practices of the participant regarding hepatitis B. Although the majority of scientific studies investigating the vaccination coverage of nursing students regarding HBV are cross-sectional, in this type of study, it cannot be determined whether the determinant precedes or follows the outcome in time. There were also limitations in the sampling technique, as the convenience sample does not allow the generalization of the conclusions of the present study to the entire population of nursing students. Regarding the tool used in the present research, although standardized, it was a self-report questionnaire and therefore has the limitations of all subjective evaluations. Also, social desirability bias may have influenced participants’ responses, particularly regarding sensitive topics such as vaccination attitudes and practices. To mitigate this, we ensured anonymity and confidentiality throughout the study and emphasized the voluntary nature of participation. The presence of the main researcher during questionnaire completion could also have influenced responses. However, we implemented measures to minimize this effect by providing clear instructions and ensuring that participants felt comfortable completing the questionnaire independently. Thus, future multicenter studies can further elucidate the above issues.

## 6. Conclusions

The present study has contributed to filling a critical gap in the literature by providing up-to-date data on HBV vaccination status, knowledge, attitudes, and practices among Greek nursing students. Our findings demonstrate that while vaccination coverage is improving, there is still significant room for enhancement. A substantial proportion of nursing students remain unvaccinated or partially vaccinated against HBV. Factors influencing vaccination status include knowledge of HBV, year of study, and attitudes and practices regarding the disease. To address these challenges, targeted interventions are necessary to improve vaccination coverage and address knowledge gaps. Additionally, this study highlights the need for further investigation into HBV vaccination practices among nursing students, as current policies may not adequately address the issue. Future research should explore the reasons for low vaccination rates and develop strategies to enhance uptake among nursing students. Furthermore, the ongoing monitoring of vaccination coverage and knowledge levels is essential to ensure the effectiveness of public health initiatives. These findings can serve as a foundation for future research and inform the development of effective public health interventions.

## Figures and Tables

**Table 1:** Socio-demographic characteristics of nursing students.

Socio-Demographic Characteristics	N (%)
Gender	
Male	267 (21.2)
Female	993 (78.8)
Year of study	
1st year	314 (24.9)
2nd year	274 (21.7)
3rd year	356 (28.3)
4th year	287 (22.8)
Greater year	29 (2.3)
Ever injured with needle	252 (20.0)

**Table 2:** Knowledge of nursing students on HBV.

Questions Regarding Knowledge on HBV	N (%)	N (%)	N (%)	Correct Answer (%)
What is the international abbreviation for hepatitis B virus?				
HBV	*926 (73.5)*			73.5
HIV	30 (2.4)			
HBC	24 (1.9)			
HPV	174 (13.8)			
I don’t know/I don’t answer	106 (8.4)			
	**No**	**Yes**	**Do not know**	
Is there a vaccine for hepatitis B virus?	28 (2.2)	*1155 (91.7)*	77 (6.1)	91.7
Is hepatitis B not common?	*688 (54.6)*	330 (26.2)	242 (19.2)	54.6
Is hepatitis B a transient infection?	*872 (69.2)*	155 (12.3)	233 (18.5)	69.2
Can people with hepatitis B spread the disease even if they feel well?	44 (3.5)	*1058 (84)*	157 (12.5)	84.0
Can people with hepatitis B spread the disease after symptoms develop?	92 (7.3)	*982 (78)*	185 (14.7)	78.0
Are healthcare professionals less vulnerable to hepatitis B infection than the general population?	*1100 (87.3)*	77 (6.1)	83 (6.6)	87.3
Is hepatitis B 100 times more infectious than HIV/AIDS?	138 (11)	*454 (36)*	668 (53)	36.0
Are infected individuals asymptomatic during the acute phase of infection?	*454 (36)*	269 (21.3)	537 (42.6)	36.0
Is hepatitis B diagnosed by taking a medical history?	*662 (52.6)*	299 (23.7)	298 (23.7)	52.6
Is hepatitis B diagnosed with the ELISA test?	83 (6.6)	*336 (26.7)*	841 (66.7)	26.7
Is hepatitis B diagnosed by measuring hepatitis markers?	57 (4.5)	*747 (59.3)*	456 (36.2)	59.3
Is hepatitis B treatment antiviral?	208 (16.5)	*500 (39.7)*	552 (43.8)	39.7
Is immunotherapy the treatment for hepatitis B?	236 (18.7)	*360 (28.6)*	664 (52.7)	28.6
Is vaccination the cure for hepatitis B?	*684 (54.3)*	312 (24.8)	264 (21)	54.3
Does hepatitis B post-exposure prophylaxis include vaccination?	472 (37.5)	*438 (34.8)*	350 (27.8)	34.8
Does hepatitis B post-exposure prophylaxis include administration of hyperimmune globulin?	68 (5.4)	*533 (42.3)*	659 (52.3)	42.3
Does hepatitis B post-exposure prophylaxis include antiviral drugs?	177 (14)	*521 (41.3)*	562 (44.6)	41.3
Does hepatitis B prevention include vaccination?	20 (1.6)	*1148 (91.1)*	92 (7.3)	91.1
Does the prevention of hepatitis B include following infection control guidelines?	53 (4.2)	*1136 (90.2)*	71 (5.6)	90.2
Does the prevention of hepatitis B include the controlled transfusion of blood and derivatives?	47 (3.7)	*1051 (83.4)*	162 (12.9)	83.4
How is hepatitis B transmitted?	No	Yes	Not sure	
Sexual intercourse	67 (5.3)	*1090 (86.5)*	103 (8.2)	86.5
From infected mother to child (vertical transmission)	21 (1.7)	*1070 (84.9)*	169 (13.4)	84.9
Contact with contaminated blood (contaminated needle, etc.)	15 (1.2)	*1208 (95.9)*	37 (2.9)	95.9
Breathing	*1056 (83.9)*	37 (2.9)	165 (13.1)	83.9
Saliva	*652 (51.7)*	311 (24.7)	297 (23.6)	51.7
Feces	*511 (40.6)*	367 (29.1)	382 (30.3)	40.6
Insect bite	*593 (47.1)*	211 (16.7)	456 (36.2)	47.1
Food	*868 (68.9)*	121 (9.6)	271 (21.5)	68.9
Sweat	*830 (65.9)*	136 (10.8)	294 (23.3)	65.9
Handshake	*1031 (81.8)*	57 (4.5)	172 (13.7)	81.8
Use of a contaminated toilet	*374 (29.7)*	501 (39.8)	385 (30.6)	29.7

Note: Correct answers are with *italics*.

**Table 3:** Attitudes and practices of nursing students on HBV.

	Mean (SD)	Median (IQR)
Should patients with hepatitis B be allowed to work? ^1^	3.4 (1.4)	4 (3–5)
Should physical exercise be allowed in patients with hepatitis B? ^1^	3.9 (1.2)	4 (3–5)
Should unprotected sex be allowed in patients with hepatitis B? ^1^	1.3 (0.7)	1 (1–1)
Should patients with hepatitis B be isolated? ^1^	2 (1.2)	2 (1–3)
Should patients with hepatitis C be vaccinated against hepatitis B? ^1^	3.7 (1.4)	4 (3–5)
Should newborns of mothers with hepatitis B be vaccinated against HBV? ^1^	4 (1.3)	5 (3–5)
Should patients with hepatitis B be hospitalized throughout their treatment? ^1^	2.4 (1.1)	2 (2–3)
After a needle-stick injury, what should you do?	**N (%)**	
Vaccinate against HBV	268 (21.3)	
Inject hyperimmune globulin	499 (39.6)	
Report to supervisor	1049 (83.2)	
I don’t know/I don’t answer	96 (7.6)	
Do you avoid nursing people with hepatitis B? ^1^	1.7 (1.0)	1.0 (1–2)
Have you participated in an educational program related to HBV in the past 12 months? ^1^	1.3 (0.8)	1.0 (1–1)
Do you consider nursing patients with hepatitis B a problem? ^1^	2.0 (1.1)	2.0 (1–3)
Do you use clean gloves in handling patients’ bodily fluids? ^1^	4.7 (0.8)	5.0 (5–5)
If you answered “Yes” in the above question, please explain why:	**N (%)**	
Personal security	944 (80.6)	
Insufficient knowledge	37 (3.2)	
Inadequate protective measures	26 (2.2)	
High risk of infecting other people	164 (14)	

^1^ Participants’ attitudes and practices towards HBV, as measured by a 5-point Likert scale (1 = strongly disagree, 5 = strongly agree). Higher scores indicate more positive attitudes and practices.

**Table 4:** Multiple logistic regression results with having been fully vaccinated against HBV in a stepwise method.

	OR (95% CI) +	*p*
Gender (female vs. male)	1.57 (1.11–2.22)	**0.011**
Third year of nursing studies vs. First year of nursing studies	1.69 (1.13–2.54)	**0.011**
Should hepatitis B patients be allowed to work? ^1^ (strongly agree vs. strongly disagree)	0.86 (0.78–0.94)	**0.002**
Do you consider hospitalizing patients with hepatitis B a problem? ^1^ (strongly agree vs. strongly disagree)	1.13 (1.01–1.28)	**0.047**
Ever injured with needle (Yes vs. No)	1.55 (1.14–2.11)	**0.013**
Knowledge score about hepatitis B	1.38 (1.25–1.52)	**<0.001**

^1^ answer could range from 1 (completely disagree) to 5 (completely agree), + odds ratio (95% confidence interval).

**Table 5:** Results from linear regression analysis in a stepwise method with dependent variable for HBV attitudes and practices score.

	β +	SE ++	B ÷	*p*
**Year of Study**				
2nd year	0.026	0.011	0.079	**0.018**
3rd year	0.013	0.011	0.042	0.246
4th or greater year	0.021	0.012	0.068	0.068
**Knowledge score regarding HBV**	0.002	0.000	0.271	**<0.001**

Note: + dependence coefficient, ++ standard error, ÷ standardized coefficient.

## Data Availability

The data presented in this study are available on reasonable request from the corresponding author due to ethical requirements.

## Public Involvement Statement

There was no public involvement in any aspect of this research.

## Guidelines and Standards Statement

This manuscript was drafted against the STROBE reporting guidelines for cross-sectional research. A complete list of reporting guidelines can be accessed via the equator network: https://www.equator-network.org/, accessed on 1 June 2024.
